# Effect of Monoacylglycerol Lipase Inhibition on Intestinal Permeability of Rats With Severe Acute Pancreatitis

**DOI:** 10.3389/fphar.2022.869482

**Published:** 2022-04-12

**Authors:** Jing Wang, Hongwei Xu, Tianjie Chen, Changqin Xu, Xiaohua Zhang, Shulei Zhao

**Affiliations:** Shandong Provincial Hospital Affiliated to Shandong First Medical University, Jinan, China

**Keywords:** monoacylglycerol lipase, severe acute pancreatitis, intestinal permeability, differentially expressed genes, alternative splicing events, RNA-binding protein

## Abstract

**Background:** Endocannabinoid 2-arachidonoylglycerol (2-AG) is an anti-nociceptive lipid that is inactivated through cellular uptake and subsequent catabolism by monoacylglycerol lipase (MAGL). In this study, we investigated the effects of MAGL inhibition on intestinal permeability and explored the possible mechanism.

**Methods:** A rat model of severe acute pancreatitis (SAP) was established. Rats were divided into three groups according to treatment. We analyzed intestinal permeability to fluorescein isothiocyanate-dextran and the levels of inflammatory factors interleukin-6 (IL-6) and tumor necrosis factor-α (TNF-α) and 2-AG. Hematoxylin and eosin staining was used to assess histological tissue changes. *In vivo* intestinal permeability was evaluated by transmission electron microscopy. We obtained ileum tissues, extracted total RNA, and applied RNA-sequencing. Sequencing data were analyzed by bioinformatics.

**Results:** Inflammatory factor levels were higher, while 2-AG levels were lower in the SAP group compared with the control group. Administration of JZL184 to rats with SAP increased the levels of 2-AG and lowered the levels of IL-6 and TNF-α. Notably, intestinal permeability was improved by JZL184 as demonstrated by fluorescein isothiocyanate-dextran measurement, hematoxylin and eosin staining, and transmission electron microscopy. RNA-sequencing showed significant transcriptional differences in SAP and JZL184 groups compared with the control group. KEGG analysis showed that the up- or downregulated genes in multiple comparison groups were enriched in two pathways, focal adhesion and PI3K-Akt signaling pathways. Differential alternative splicing (AS) genes, such as Myo9b, Lsp1, and Git2, have major functions in intestinal diseases. A total of 132 RNA-binding proteins (RBPs) were screened by crossing the identified abnormally expressed genes with the reported RBP genes. Among them, HNRNPDL coexpressed the most AS events as the main RBP.

**Conclusion:** MAGL inhibition improved intestinal mucosal barrier injury in SAP rats and induced a large number of differentially expressed genes and alternative splicing events. HNRNPDL might play an important role in improving intestinal mucosal barrier injury by affecting alternative splicing events.

## Introduction

Acute pancreatitis is an unpredictable and potentially fatal disease. Patients with acute pancreatitis often develop severe systemic inflammatory diseases, resulting in systemic impairment of multiple organ functions, which greatly increases mortality and treatment difficulty (Boxhoorn et al., 2020). The intestinal tract is an important organ that is damaged by severe acute pancreatitis (SAP). Injury of the intestinal mucosal barrier can also aggravate SAP. The injury of the intestinal mucosal barrier during SAP may be related to microcirculation disturbance, excessive release of inflammatory mediators, ischemia–reperfusion injury, and intestinal epithelial cell apoptosis. Therefore, exploring the molecular mechanism of apoptosis, inflammatory response, and oxidative stress in the process of intestinal mucosal barrier injury in SAP may facilitate finding therapeutic drugs (Ge et al., 2020; Li et al., 2020).

Monoacylglycerol lipase (MAGL) is a serine hydrolase that hydrolyzes 2-arachidonoylglycerol (2-AG), a component of the endogenous cannabinoid system. MAGL plays a major role in gastrointestinal diseases. Cannabinoids and the cannabinoid receptor, have major functions in pancreatitis. Activating or inhibiting the cannabinoid receptor affects the development of pancreatitis (Dembiński et al., 2006; Michalski et al., 2007; Zyromski et al., 2009; Michler et al., 2013).

MAGL inhibitors regulate the inflammatory response and intestinal barrier, and are effective therapeutic drugs (Alhouayek and Muccioli, 2012; Maselli and Camilleri, 2021). Numerous MAGL inhibitors are available, including MJN110, KML29, URB602, SAR127303, OMDM169, and ABX1431 (Granchi et al., 2017). JZL184, a piperidine carbamate that preferentially and irreversibly inhibits MAGL, was the first pharmacological drug to acutely increase levels of 2-AG in the brain without altering levels of AEA. Thus, JZL184 was the first selective MAGL inhibitor (Long et al., 2009). There is a growing body of research demonstrating that inhibition of MAGL reduces nociceptive behavior in animal models of neuropathic pain (Wilkerson et al., 2016; Kamimura et al., 2018).

At present, there are no report on MAGL in SAP. The function and mechanism of MAGL inhibitors in intestinal mucosal barrier injury caused by SAP are unclear, requiring further exploration.

## Materials and Methods

### Animals

We obtained male SD rats (200–230 g) from the Experimental Animal Center of Shandong University (Jinan, Shandong, China). The SD rats were raised in an animal facility at 22°C, and with an automatic 12-h light/dark cycle. All the animal experiments were authorized by the Shandong Provincial Hospital Committee on Use and Care of Animals. The researchers in this study were blinded to details of animal treatments.

### SAP Rat Model Establishment and Protocol of Experiments

We used a well-established protocol to generate the rat model of SAP. SD rats, 6–8 weeks of age, were anesthetized, and a median incision was made to locate the duodenal opening. A No. 5 puncture needle was used to retrogradely puncture the pancreaticobiliary duct near the duodenal opening. The puncture needle at the duodenal opening and the hilar opening of the common bile duct was fixed in place with two non-invasive artery clamps. A micropump was used to inject 3% sodium taurocholate solution into the pancreatic duct at a uniform rate of 0.1 ml/min (at a dose of 0.1 ml/100 g). The needle was maintained in place for 5 min after injection, and after visually observing that the color of the pancreas turned dark red, the puncture needle was pulled out, the vascular clamp was loosened, and the duodenal wall puncture was sutured to avoid intestinal fluid leakage into the abdominal cavity. After confirming that no active bleeding was observed, the abdominal cavity was closed layer by layer.

### Animal Grouping and Sample Collection

The housed SD rats were equally and randomly divided into three groups 24 h after the surgery: Control group, SAP group and SAP treated with JZL184 group. The JZL184 group was received intraperitoneal injection of the MAGL inhibitor JZL184 (Cayman Europe, Talin, Estonia), which was prepared in a mixture of saline/ethanol/Tween-80 and administered at a dosage of 10 mg/kg. At the same time, in the Control and SAP groups, the SD rats were intraperitoneally injected with the same volume of vehicle. After 24 h of intervention, the rats were killed for further experiments. We collected blood samples from the inferior vena cava and centrifuged them at 3,000 g at 4°C for 5 min. The supernatant of blood samples was then stored at −20°C for subsequent serum analysis. The ileal tissue adjacent to the cecum was removed and then fixed with 4% paraformaldehyde for sectioning; the other tissues were stored at −80°C for later experiments.

### 2-AG Quantification

Quantification of 2-AG was performed with high-performance liquid chromatography-mass spectrometry (HPLC-MS), following our previous study protocol (Wang et al., 2020).

### *In Vivo* Measurement of FITC-Dextran Permeability

Experimental rats were treated with 4 kDa fluorescein isothiocyanate-dextran (FD4, 400 mg/kg) in phosphate-buffered saline (PBS, pH 7.4) by gavage. Blood was then collected by heart puncture after 4 h. The blood samples were then centrifuged at 1,000 g for 5 min, and stored at −80°C until analysis. To measure FD4 levels, 25 μl serum was diluted with 175 μl PBS in a black-wall microplate, and fluorescence values were compared with the serial dilutions of known FD4 concentrations. All measurements were carried out in triplicate to eliminate experimental errors.

### IL-6 and TNF-α Measurements

Arterial blood was collected 24 h after treatment. IL-6 and TNF-α levels in serum were measured by ELISA method. IL-6 and TNF-α kits were purchased from Bioswamp (Wuhan, China). All procedures were performed based on the manufacturer’s instructions. The OD values were obtained on a microplate reader (450 nm), and the corresponding concentrations of TNF-α and IL-6 were calculated and recorded. Measurements were carried out in triplicate.

### Histopathological Evaluation

We fixed ileal tissues with formalin (4%) and embedded them in paraffin for histological analysis. Tissue sections (5 μm) were then stained with hematoxylin and eosin (HE). We used a light microscope to observe the morphological changes. The injury degree to the ileum was scored based on previously published criteria (Chiu et al., 1970).

### Transmission Electron Microscopy

*In vivo* intestinal permeability was assessed by TEM. We washed fresh ileal tissue with precooled PBS and then fixed them in 1% osmium tetroxide and 2.5% phosphate-buffered glutaraldehyde. We then used an ultra-microtome to section the ileal tissue samples and stained them with uranyl acetate and lead citrate. Ultrastructural images of intestinal epithelial cells (IECs) were then captured by TEM (Hitachi HT7700, Japan).

### RNA Extraction and Sequencing

Total RNAs were extracted from ileal tissue of rats with TRIzol Reagent (Invitrogen, NO 15596026) adopting the methods by Chomczynski and Sacchi, 1987. Then DNA digestion was conducted by DNaseI. After analyzing A260/A280 with NanodropTM OneCspectrophotometer (Thermo Fisher Scientific Inc), RNA quality was determined. RNA Integrity was assessed using 1.5% agarose gel electrophoresis. Qubit3.0 with QubitTM RNA Broad Range Assay kit (Life Technologies, Q10210) were used to quantify the qualified RNAs. About 2 μg RNAs per sample were used for stranded transcriptome library preparation using KCTM Stranded mRNA Library Prep Kit for Illumina (Catalog NO. DR08402, Wuhan Seqhealth Co., Ltd. China) under the instruction. PCR products with 200–500 bps were enriched, quantified and finally sequenced on Novaseq 6,000 sequencer (Illumina) with PE150 model.

### RNA-Seq Raw Data Cleaned and Aligned

We first discarded raw reads with unknown N bases. Sequencing adaptors and low-quality bases were then trimmed following the first step by FASTX-Toolkit (Version 0.0.13). Too short reads (less than 16 nt) were also removed. Quality filtered reads were then aligned onto the mRatBN7.2 genome using HISAT2 (Kim et al., 2013) with no more than four mismatches. Uniquely aligned reads were used to count the number of gene reads and to calculate FPKM (fragments per kilobase of transcript per million fragments mapped) (Trapnell et al., 2010).

### Differentially Expressed Genes Analysis

The R Bioconductor package DESeq2 (Love et al., 2014) was used to identify the differentially expressed genes (DEGs). The *p* value after correction <0.05 and fold change > 2 or < 0.5 were set as the threshold for the identification of DEGs.

### Alternative Splicing Analysis

As described previously, the alternative splicing events (ASEs) and regulated alternative splicing events (RASEs) were defined and quantified using the ABLas pipeline (Jin et al., 2017; Xia et al., 2017). Briefly, ABLas detection of ten types of ASEs was based on the splicing junction reads, including exon skipping (ES), alternative 5' splice site (A5SS), alternative 3'splice site (A3SS), mutually exclusive exons (MXE), mutually exclusive 5'UTRs (5pMXE), mutually exclusive 3'UTRs (3pMXE), cassette exon, A3SS&ES and A5SS&ES. To predict RASEs, Student’s *t*-test was used to assess the significant *p*-values of the ratio difference for AS events. AS events with 0.05 false discovery rate based on *p*-value were regarded as RASEs.

### Functional Enrichment Analysis

To analyze functional categories of DEGs, Gene Ontology (GO) and kyoto encyclopedia of genes and genomes (KEGG) pathway enrichment analysis was performed by KOBAS 2.0 server (Xie et al., 2011). Hyper geometric test and false discovery rate controlling procedure were carried out to define the significance of each pathway.

### Real-Time Quantitative Reverse Transcription Validation of DEG and AS

To validate the DEGs and RASEs from RNA-seq result, RT-qPCR was conducted using GAPDH as a reference. RT-qPCR conditions were prepared as follows: denaturation was under 95°C for 5 min, 40 cycles of denaturing process was carried out at 95°C for 15 s, then with annealing and extension at 60°C for 30 s. Three technical replicates for each sample were prepared for experiment. The primers used for experiment were listed in Supplementary Table S1.

### Other Statistical Analyses

To show the sample clustering with the first two components, principal component analysis (PCA) was conducted using the R package factoextra (https://cloud.r-project.org/package=factoextra). In house tool Sogen was utilized to view the RNA-seq data with gene structure and junction reads. Clustering analysis and presentation were carried out by R heatmap package (https://cran.r-project.org/web/packages/pheatmap/index.html). We used Student’s *t*-test to compare the statistical significance between two groups.

### Availability of the Raw RNA-Seq Data

The raw RNA-seq data analyzed in this research are available under GEO Series accession number GSE 197930.

## Results

1. JZL184 protect SAP rats from intestinal permeability injury

**FIGURE 1:**
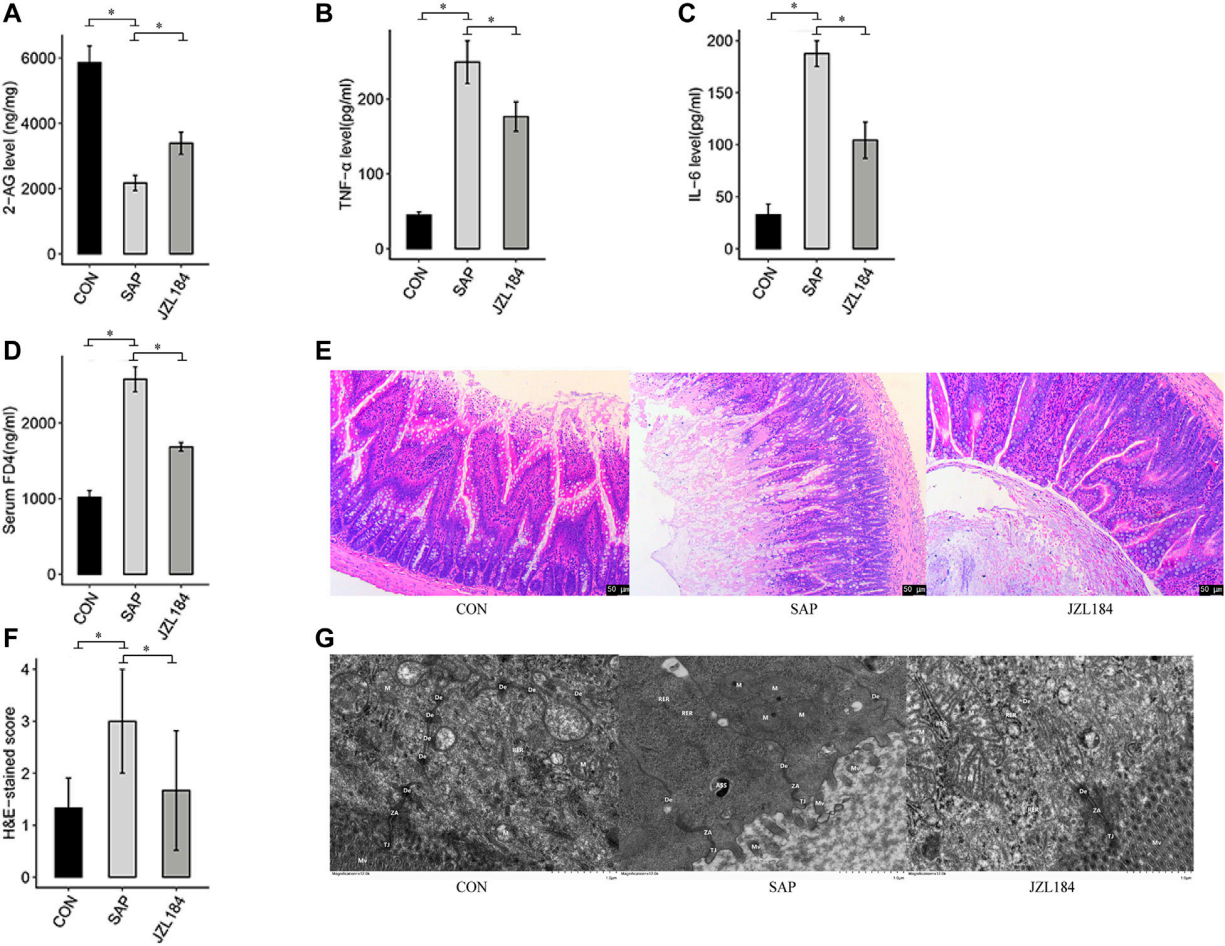
MAGL inhibitor affect intestinal permeability of rats with SAP. **(A)** 2-AG levels detected in each group. The levels of 2-AG were significantly lower in the SAP group than in the control group (*p* < 0.05) and JZL184 partially restored 2-AG levels in the SAP group; **(B)** IL-6 levels detected in each group. The concentrations of IL-6 was higher in the SAP group than in the control group (*p* < 0.05). JZL184 decreased IL-6 levels; **(C)** TNF-α levels detected in each group. The concentrations of TNF-α was higher in the SAP group than in the control group (*p* < 0.05). JZL184 decreased TNF-α levels; **(D)** FD4 concentrations detected in each group. The FD4 concentration was higher in the SAP group than in the control group (*p* < 0.05). JZL184 decreased serum FD4 levels (*p* < 0.05); **(E)** Representative HE-stained images of ileal sections in each group (original magnification, ×100). No obvious intestinal mucosal injury was found in the control group. However, there were signs of intestinal injury in the SAP group; **(F)** Pathological scores for the ileum in each group. The intestinal tissues of SAP rats treated with JZL184 displayed a lesser degree of morphological changes and lower pathological scores than those of the SAP group (*p* < 0.05); **(G)** The ultrastructure of IECs observed by TEM in each group. No obvious intestinal barrier damage was found in the control group, whereas the intestinal barrier was severely damaged in the SAP group. These histopathological changes were ameliorated by JZL184. *n* = 3 in each group. *p < 0.05, **p < 0.01.

We established a rat model of SAP and examined the effect of JZL184. HPLC-MS analysis showed that the levels of 2-AG in the tissues of the ileum were significantly lower in the SAP group compared with the control group, and that JZL184 partially restored 2-AG levels in the SAP group (Figure 1A). The serum levels of TNF-α and IL-6 were measured by ELISAs. The concentrations of TNF-α and IL-6 were higher in the SAP group than in the control group (*p* < 0.05). Administration of JZL184 decreased TNF-α and IL-6 levels (Figures 1B,C).

We next investigated the role of JZL184 in intestinal permeability. The FD4 concentration was higher in the SAP group than in the control group (*p* < 0.05). Notably, the concentration of serum FD4 was significantly lower in the JZL184 group than in the SAP group (*p* < 0.05) (Figure 1D). Histological changes in intestinal mucosal tissues were evaluated by hematoxylin and eosin (HE) staining. No obvious intestinal mucosal injury was found in the control group. As shown in Figure 1E, there were signs of intestinal injury in the SAP group, which included edema, villus breakage, exposure of lamina propria capillaries, and an increase in inflammatory cells. The intestinal tissues of SAP rats treated with JZL184 displayed a lesser degree of morphological changes than those of the SAP group (*p* < 0.05) (Figure 1F). TEM revealed no obvious intestinal barrier damage in the control group, whereas the intestinal barrier was severely damaged in the SAP group with sparse and fractured microvilli, fewer intercellular connections, sparse desmosome tension filaments, and local widening of the intercellular space. These histopathological changes were ameliorated by JZL184 (Figure 1G).

2. JZL184 broadly affects the gene expression profile of intestinal tissue in rats with SAP

**FIGURE 2:**
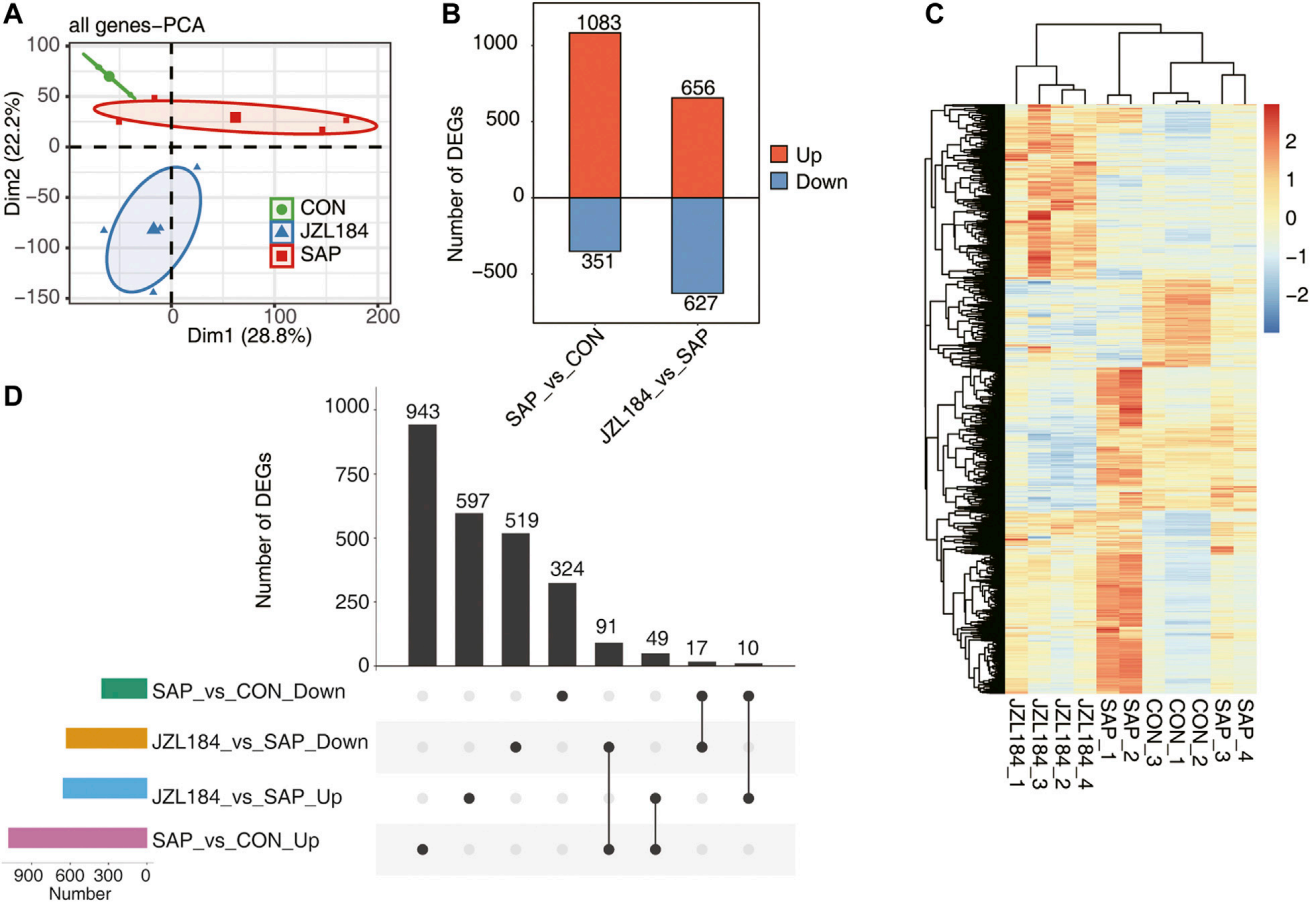
MAGL inhibitor broadly affect the gene expression profile of intestinal tissue of rats with SAP. **(A)** PCA base on FPKM value of all detected genes. The ellipse for each group is the confidence ellipse. The confidence ellipsis of the three groups did not overlap and the samples were obviously separated; **(B)** Bar plot showing the result of up-regulated and downregulated genes in the group comparisons. DEG analysis showed that 1,434 genes were differentially expressed between SAP and control groups; **(C)** Hierarchical clustering heat map showing expression levels of all DEGs. The expression of DEGs in SAP group showed slight differences due to individual responses to experimental treatments; **(D)** Overlap analysis plots of DEGs genes in each group. A total of 91 DEGs were downregulated in the JZL184 group compared with the SAP group, but were upregulated in the SAP group compared with the control group. *n* = 3 in control group. *n* = 4 in SAP group and JZL184 group.

The mRNA expression profile of intestinal mucosa is presented in Figure 2. To explore sample clustering among the three groups, we performed PCA in accordance with the expression levels of all detected genes. The results showed that the confidence ellipsis of the three groups did not overlap and the samples were obviously separated (Figure 2A). Differentially expressed gene (DEG) analysis showed that 1,434 genes were differentially expressed between SAP and control groups (FC ≥ 2 or ≤0.5, FDR<0.05), of which 1,083 genes were upregulated and 351 genes were downregulated in the SAP group. Moreover, 1,283 genes were differentially expressed between JZL184 and SAP groups, of which 656 genes were upregulated and 627 genes were downregulated in the JZL184 group (Figure 2B).

An expression heat map of differentially expressed genes (DEGs) showed that three samples of control and JZL184 groups were gathered together, while four samples of the SAP group were divided into two groups. The expression of DEGs in samples of the SAP group showed slight differences due to individual responses to experimental treatments (Figure 2C). In differential gene overlap analysis of each group, 943 DEGs were specifically upregulated in the SAP group compared with the control group, 597 DEGs were specifically upregulated in the JZL184 group compared with the SAP group, 519 DEGs were specifically downregulated in the JZL184 group compared with the SAP group, and 324 DEGs were specifically downregulated in the SAP group compared with the control group. A total of 91 DEGs were downregulated in the JZL184 group compared with the SAP group, but were upregulated in the SAP group compared with the control group (Figure 2D).

3. Functional analysis of MAGL inhibitor-regulated DEGs in intestinal tissue of rats with SAP

**FIGURE 3:**
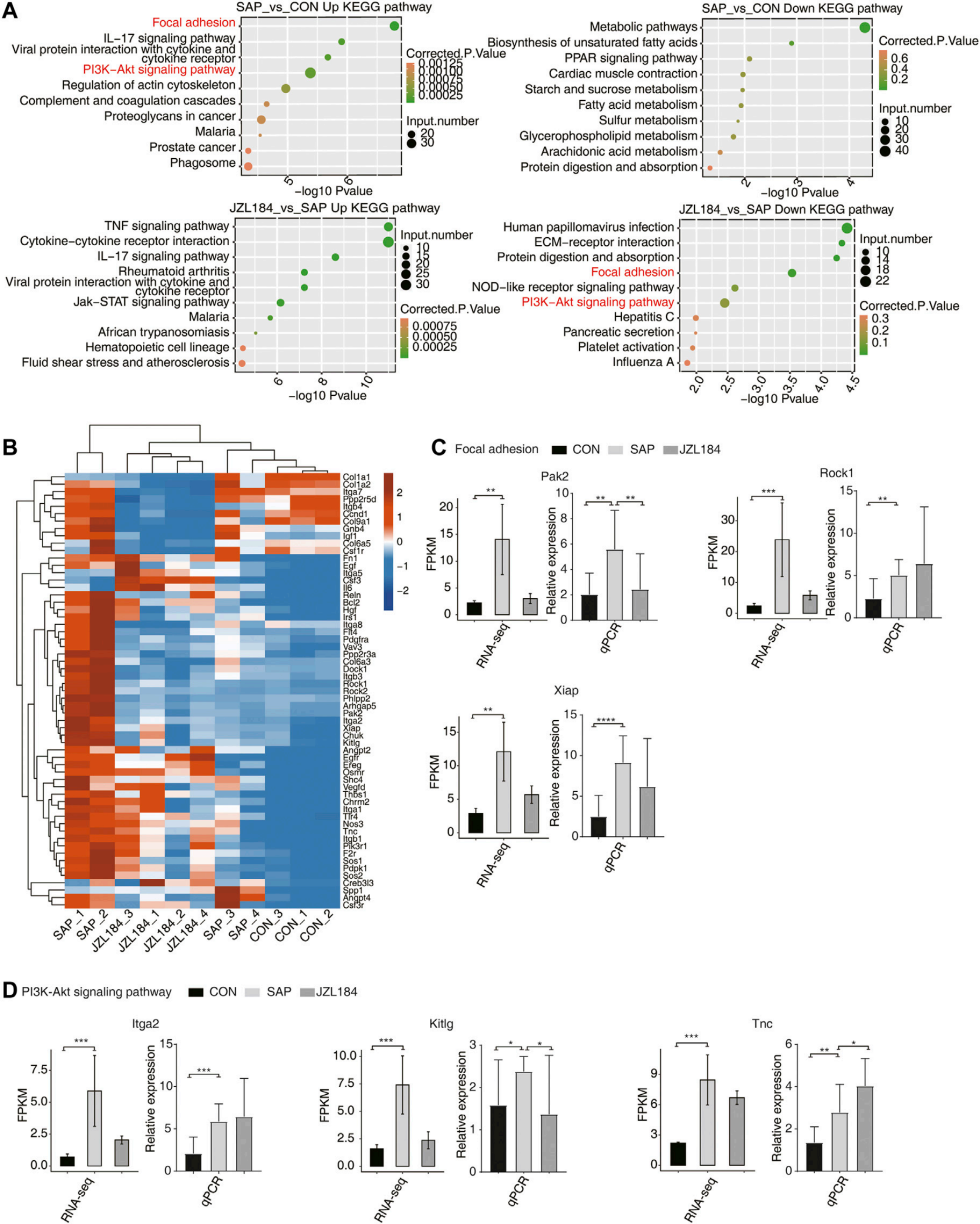
MAGL inhibitor extensively change alternative splicing pattern of genes in intestinal tissue of rats with SAP. **(A)** Bubble plot showing the top ten enriched KEGG terms for up regulated (Left panel) and down regulated (Right panel) DEGs. The downregulated genes in the SAP group were mainly enriched in human papillomavirus infection, ECM receptor interaction, protein digestion and absorption, focal adhesion, nod-like receptor signaling pathway, PI3K-Akt signaling pathway, hepatitis C, pancreatic secretion, platelet activation, and influenza **(A,B)**. Hierarchical clustering heat map showing the expression levels of all genes on Focal adhesion and PI3 K-Akt signaling pathway in Top 10 in the KEGG analysis. The expression of Rock1, Rock2, Pak2, Xiap, Arhgap5, Itga2, Tnc, Dock1, Col6a3, Pdgfra, Chuk, Kitlg, Sos1, Ppp2r3a, and Phlpp2 was higher in the SAP group and lower in the JZL184 group; **(C)** Bar plot showing the expression pattern and statistical difference of DEGs for some important genes in Focal adhesion. Error bars represent mean ± SEM. The expression of Pak2, Rock1, and Xiap was increased in the SAP group compared with the control group, which was consistent with our expectations. Moreover, expression of Pak2 and Xiap was decreased in the JZL184 group compared with the SAP group; ***p-value < 0.001, **p-value < 0.01. **(D)** Bar plot showing the expression pattern and statistical difference of DEGs for some important genes in PI3K-Akt signaling pathway. Error bars represent mean ± SEM. The expression of Itga2, Kitlg, and Tnc was increased in the SAP group and that of Kitlg was decreased compared with the control group, whereas Itga2 and Tnc gene expression was increased in the JZL184 group compared with the SAP group. *** *p*-value < 0.001, ** *p*-value < 0.01. *n* = 3 in control group. *n* = 4 in SAP group and JZL184 group.

On the basis of the heat map of DEGs, we further examined the genes upregulated in the SAP group, as well as the weakly expressed genes in the JZL184 and control groups. KEGG analysis of up- and downregulated genes in each group showed that, when comparing the SAP and control groups, upregulated genes in the SAP group were mainly related to focal adhesion; the IL-17 signaling pathway; interactions between viral proteins, cytokines, and cytokine receptors; the PI3K-Akt signaling pathway; regulation of the actin cytoskeleton; the complement and aggregation cascade; and proteoglycans in malaria, prostate cancer, and phagosomes. When comparing the JZL184 and SAP groups, KEGG showed that the downregulated genes in the SAP group were mainly enriched in human papillomavirus infection, ECM receptor interaction, protein digestion and absorption, focal adhesion, nod-like receptor signaling pathway, PI3K-Akt signaling pathway, hepatitis C, pancreatic secretion, platelet activation, and influenza A (Figure 3A).

We extracted all genes from focal adhesion and PI3K-Akt signaling pathways among the top 10 in the KEGG analysis, and then we combined them and drew a heat map of the DEGs (Figure 3B). Compared with the control group, expression of Rock1, Rock2, Pak2, Xiap, Arhgap5, Itga2, Tnc, Dock1, Col6a3, Pdgfra, Chuk, Kitlg, Sos1, Ppp2r3a, and Phlpp2 was higher in the SAP group and lower in the JZL184 group (Figure 3B).

Next, we performed RT-qPCR on selected DEGs using their RNA-sequencing data. In the focal adhesion pathway, the expression of Pak2, Rock1, and Xiap was increased in the SAP group compared with the control group, which was consistent with our expectations. Moreover, expression of Pak2 and Xiap was decreased in the JZL184 group compared with the SAP group. The *t*-test results showed that Pak2 was significantly different in dominance, which was consistent with expectations; Xiap had no significant difference, but the trend was the same (Figure 3C). In the PI3K-Akt pathway, compared with the control group, expression of Itga2, Kitlg, and Tnc was increased in the SAP group and that of Kitlg was decreased, whereas Itga2 and Tnc gene expression was increased in the JZL184 group compared with the SAP group. Kitlg was significantly different in dominance, which was consistent with our expectations (Figure 3D).

4. MAGL inhibition extensively changes the alternative splicing pattern of genes in the intestinal tissue of rats with SAP

**FIGURE 4:**
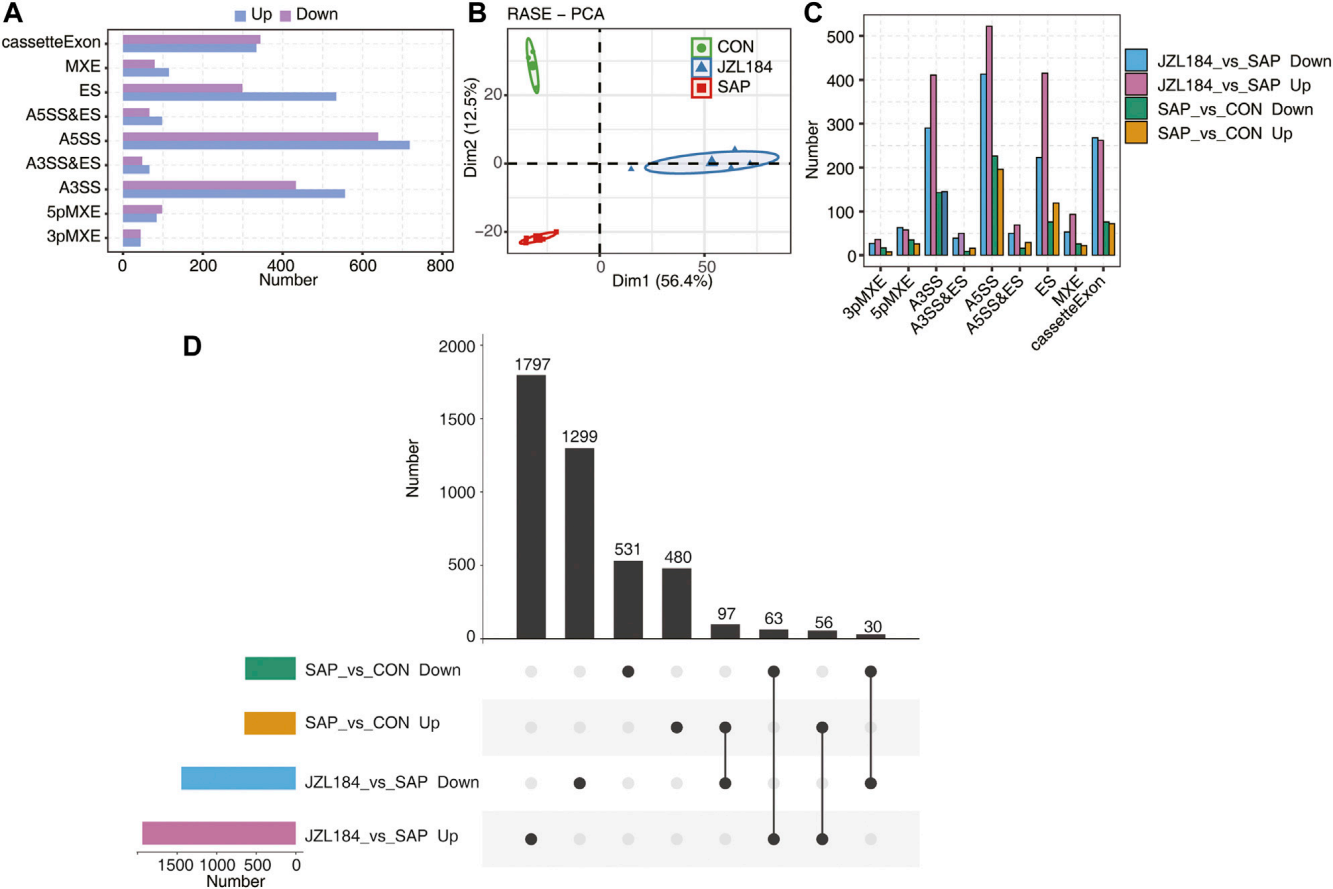
MAGL inhibitor extensively change alternative splicing pattern of genes in intestinal tissue of rats with SAP. **(A)** Bar plot showing the MAGL regulated alternative splicing event (RASE) numbers by classifying them into nine type. **(B)** PCA base on Ratio value of the MAGL regulated alternative splicing event (RASE). The ellipse for each group is the confidence ellipse. The results showed that the confidence ellipsis of the three groups did not overlap and the samples were obviously discriminated; **(C)** Bar plot showing the MAGL regulated alternative splicing event (RASE) numbers in the four comparison groups. 480 ASEs were specifically upregulated in the SAP group compared with the control group, 1797 ASEs were specifically upregulated in the JZL184 group compared with the SAP group, 1,299 ASEs were specifically downregulated in the JZL184 group compared with the SAP group, and 531 ASEs were specifically downregulated in the SAP group compared with the control group; **(D)** Overlap analysis plots of RASE in each group. 97 ASEs were downregulated in the JZL184 group compared with the SAP group, but were upregulated in the SAP group compared with the control group; *n* = 3 in control group. *n* = 4 in SAP group and JZL184 group.

On the basis of RNA-sequencing data, 63,412 alternative splicing events (ASEs) were identified in each sample, which included 27,353 known ASEs and 36,056 newly identified ASEs. A *t*-test was used to compare the changes in AS levels of the same splicing pattern of each gene in the two comparison groups. The results revealed 1,257 differential ASEs between the SAP and control groups and 3,342 differential ASEs between the JZL184 and SAP groups. The ASEs of intestinal mucosa are presented in Figure 4. MAGL-regulated alternative splicing event (RASE) numbers were classified into nine types (Figure 4A). The results showed that the confidence ellipsis of the three groups did not overlap and the samples were obviously discriminated (Figure 4B). We found that 480 ASEs were specifically upregulated in the SAP group compared with the control group, 1797 ASEs were specifically upregulated in the JZL184 group compared with the SAP group, 1,299 ASEs were specifically downregulated in the JZL184 group compared with the SAP group, and 531 ASEs were specifically downregulated in the SAP group compared with the control group (Figure 4C). Overlap analysis showed that 97 ASEs were downregulated in the JZL184 group compared with the SAP group, but were upregulated in the SAP group compared with the control (Figure 4D).

5. Functional analysis of MAGL inhibitor-regulated alternative genes in intestinal tissue of rats with acute pancreatitis

**FIGURE 5:**
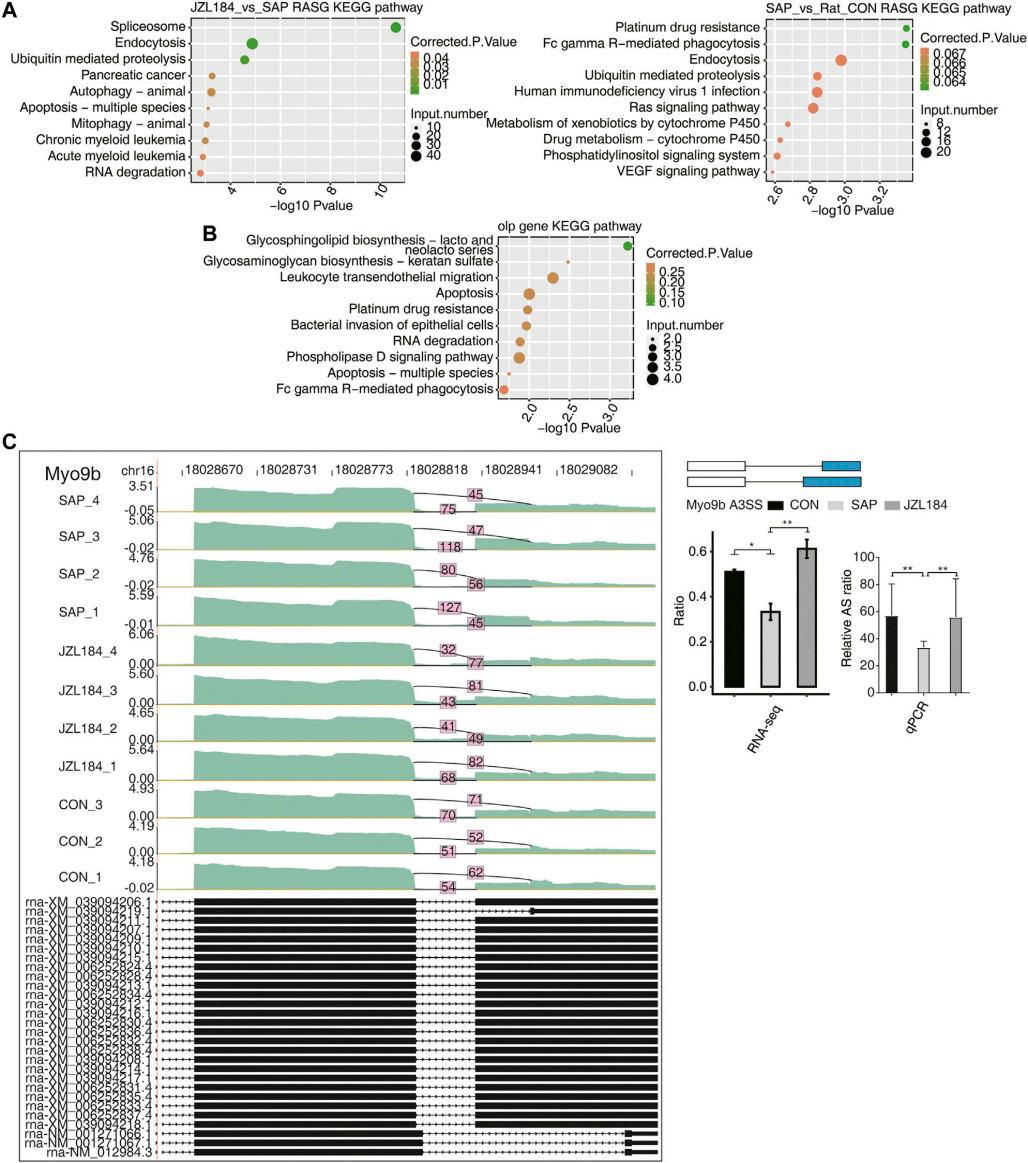
Function analysis of MAGL inhibitor regulated alternative genes in intestinal tissue of rats with SAP. **(A)** Bubble plot showing enriched KEGG analysis plots of RASE. The differential ASEs were mainly enriched in platinum resistance, FC-γR-mediated phagocytosis, endocytosis, ubiquitin-mediated proteolysis, human immunodeficiency virus 1 infection, Ras signaling pathway, cytochrome P450 metabolism of exogenous drugs, drug metabolism cytochrome P450, phosphatidylinositol signaling system, and the VEGF signaling pathway between the SAP and control groups; AS genes were mainly enriched in splicing, endocytosis, ubiquitin-mediated proteolysis, pancreatic cancer, animal autophagy, apoptosis of many species, animal mitochondrial autophagy, chronic myelogenous leukemia, acute myeloid leukemia, RNA degradation, and other functional pathways between the JZL184 and SAP groups; **(B)** Bubble plot showing enriched KEGG analysis of the genes with overlapped shear events in the opposite direction obtained by the two comparison groups; **(C)** MAGL regulates alternative splicing of Myo9b. Left panel: IGV-sashimi plot showing the regulated alternative splicing events and binding sites across mRNA. Reads distribution of RASE is plotted in the up panel and the transcripts of each gene are shown below. Right panel: The schematic diagrams depict the structures of ASEs. RNA-seq validation of ASEs are shown at the bottom of the right panel. Error bars represent mean ± SEM. *** *p*-value < 0.001, ** *p*-value < 0.01, * *p*-value < 0.05. *n* = 3 in control group. *n* = 4 in SAP group and JZL184 group.

To clarify the potential functions of these differential ASEs, we extracted genes with the above RASEs and performed KEGG analysis. When comparing the SAP and control groups, the differential ASEs were mainly enriched in platinum resistance, FC-γR-mediated phagocytosis, endocytosis, ubiquitin-mediated proteolysis, human immunodeficiency virus 1 infection, Ras signaling pathway, cytochrome P450 metabolism of exogenous drugs, drug metabolism cytochrome P450, phosphatidylinositol signaling system, and the VEGF signaling pathway (Figure 5A). In the comparison between the JZL184 and SAP groups, AS genes were mainly enriched in splicing, endocytosis, ubiquitin-mediated proteolysis, pancreatic cancer, animal autophagy, apoptosis of many species, animal mitochondrial autophagy, chronic myelogenous leukemia, acute myeloid leukemia, RNA degradation, and other functional pathways (Figure 5A). A bubble plot showed enriched KEGG analysis of the genes with overlapped ASEs in the opposite direction obtained from the two comparison groups (Figure 5B).

Expression of Epb4113, Rpusd3, Myo9b, Lsp1, Git2, Cbx3, and Ar14a was decreased in the SAP group compared with the control group, but increased in the JZL184 group. We performed RT-qPCR on Myo9b, Lsp1, and Git2 from RNA-sequencing data. The *t*-test results showed that they were significantly different in dominance, which was consistent with expectations (Figure 5C, Supplementary Figure S1).

6. Coexpression analysis between MAGL inhibitor-regulated RBPs and RASEs in the intestinal tissue of rats with SAP

**FIGURE 6:**
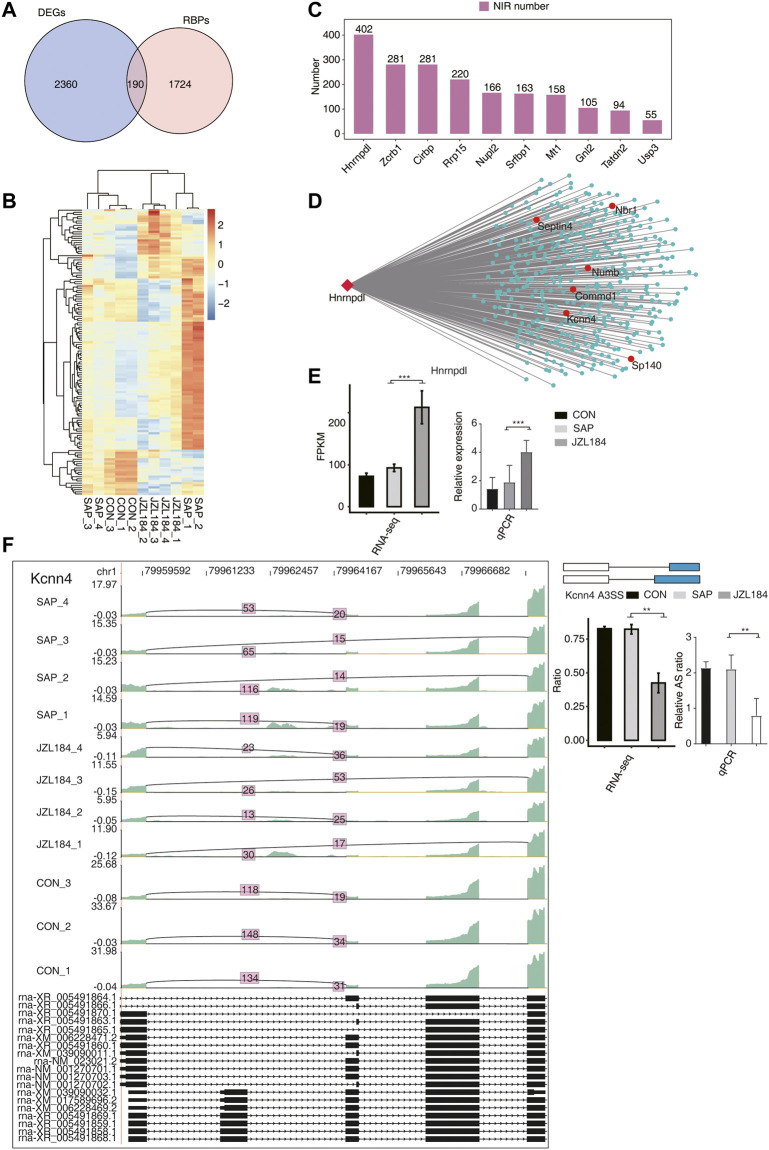
Co-expression analysis between MAGL inhibitor regulated RBPs with NIR in intestinal tissue of rats with SAP. **(A)** Venn diagram showing the overlapped genes between RBPs and DEGs; **(B)** Hierarchical clustering heat map showing the expression levels of the overlapped genes in **(A)**. Significant differences in the expression levels of these RBPs were found among the groups (Show expression FPKM greater than 1 in 80% of the samples); **(C)** Bar plot showing the number of NIR expressed showing the top10 RBPs sorted by the number of RBPs co-expressed from the above screened RBPs; **(D)** Co-expression network between NIRs and Hnrnpdl gene. These RBPs were specifically and highly expressed in the JZL184 group, in which the ASEs co-expressed by HNRNPDL comprised the most, which reached 402 ASEs; **(E)** Bar plot showing the expression pattern and statistical difference of DEGs for Hnrnpdl gene. Error bars represent mean ± SEM; ***p-value < 0.001. **(F)** MAGL regulates alternative splicing of Kcnn4. Left panel: IGV-sashimi plot showing the regulated alternative splicing events and binding sites across mRNA. Reads distribution of RASE is plotted in the up panel and the transcripts of each gene are shown below. Right panel: The schematic diagrams depict the structures of ASEs. RNA-seq validation of ASEs are shown at the bottom of the right panel. Error bars represent mean ± SEM. *** *p*-value < 0.001, ** *p*-value < 0.01, * *p*-value < 0.05. *n* = 3 in control group. *n* = 4 in SAP group and JZL184 group.

By crossing the identified DEGs with reported potential RBP genes, 190 DEGs were found to be RBPs (Figure 6A). A total of 132 RBPs with an expression level greater than 1 in more than 80% of the samples were screened according to their expression levels, and we found significant differences in the expression levels of these RBPs among the groups (Figure 6B). According to the expression level of the 132 RBPs in all samples and the ratio of different splicing isoforms supporting reads of all differential RASEs, the RASEs co-expressed by 132 RBPs were identified. In accordance with the order of the number of RASEs co-expressed by each RBP, the top 10 RBPs were extracted, which included HNRNPDL, ZCRB1, CIRBP, RRP15, NUPl2, SRFBP1, MT1, GNL2, TATDN2, and USP3 (Figure 6C). We found that these RBPs were specifically and highly expressed in the JZL184 group compared with the control and SAP groups, in which the ASEs co-expressed by HNRNPDL comprised the most, which reached 402 ASEs (Figure 6D). Figure 6E shows the verification of Hnrnpdl, and the results are consistent with the RNA-sequencing results. NIR events coexpressed with HNRNPDL were further extracted. In accordance with the number of supported reads, the proportion of reads and its change amplitude of coexpressed ASEs and the change trend of the ratio value in each group, the ratio of ASEs of Numb, Commd1, Kcnn4, Sp140, Septin4, and Nbr was consistent or opposite to the change trend of Hnrnpdl expression. RT-qPCR demonstrated a statistically significant decrease in Kcnn4 and Sp140, which was in agreement with the RNA-sequencing analysis (Figures 6F, Supplementary Figure S2).

## Discussion

The etiology of SAP remains unclear. However, recent reports suggest involvement of the endocannabinoid system in the pathophysiology of SAP (Michalski et al., 2007; Michler et al., 2013; Petrella et al., 2010; da Silva-Leite et al., 2018). Identification of MAGL activity was first described in 1966 (Kupiecki, 1966). This enzyme degrades 2-AG, the most abundant endocannabinoid in the body (Dinh et al., 2002). Strong upregulation of 2-AG has been observed in MAGL knockout mice, which implies that most endocannabinoids are degraded by hydrolysis (Taschler et al., 2015). MAGL is currently considered a promising therapeutic target for the treatment of numerous diseases that include gastrointestinal disorders, cancer, and neurodegenerative and inflammatory diseases (Grabner et al., 2017). In the present study, we found decreased 2-AG levels in the ileal tissue of rats in the SAP group compared with the control group. Thus, MAGL and 2-AG play important roles in the development of SAP and may represent potential therapeutic targets for SAP.

Activation of pancreatic proteases and dysfunction of pancreatic microcirculation in acute pancreatitis stimulate granulocytes, macrophages, and vascular endothelial cells to release IL-6, TNF-α, and other cytokines that participate in the inflammatory response and immunoregulation (Pooran et al., 2003; Sternby et al., 2021). The release of inflammatory factors not only damages local pancreatic tissue, but also leads to a systemic inflammatory response because of the massive release of inflammatory factors into the circulation, which can result in systemic organ failure. Our findings showed that the concentrations of TNF-α and IL-6 in serum were higher in the SAP group than in the control group, which indicated that they may be involved in the occurrence and development of SAP. Notably, JZL184 downregulated TNF-α and IL-6, which suggested that it had the potential to improve systemic inflammatory response syndrome and prevent multiple organ dysfunction.

Changes in mucosal permeability may occur because of mucosal damage. Several studies have demonstrated increases in intestinal permeability in patients with SAP and SAP-related infections (Schietroma et al., 2016; Ge et al., 2020). The underlying mechanisms include alterations in the expression, localization, and function of tight junction proteins as well as changes in microbiota, active inflammation, and/or proinflammatory cytokines (Schietroma et al., 2016). Under the stress state caused by SAP, the structure and function of intestinal mucosa are perturbed, which results in a pathological increase in intestinal mucosal permeability (Zhong et al., 2009; Meriläinen et al., 2013).

In our previous study, JZL184 was administered to rats subjected to chronic stress. In these animals, intestinal permeability was increased after administration of JZL184 and associated with upregulation of tight junction-associated proteins. Furthermore, the effects of MAGL inhibition were mediated by CB1 (Wang et al., 2020). Based on this finding, we investigated the effect of JZL184 on intestinal mucosal permeability in a rat model of SAP. We measured the concentration of plasma FD4, observed histological changes by HE staining, and examined changes in the IEC microstructure by TEM. All data showed that intestinal mucosal permeability was impaired by SAP and that JZL184 ameliorated the pathological changes. Furthermore, 2-AG was increased in the JZL184 group compared with the SAP group. This suggests that the increased levels of 2-AG may directly affect intestinal permeability.

Understanding the molecular mechanism of intestinal mucosal permeability is helpful for the diagnosis and treatment of SAP rats. In the current study, RNA sequencing showed that a large number of genes were differentially expressed in the ileum of the SAP and JZL184 groups compared with the control group. SAP may cause upregulated expression of focal adhesion and PI3K-Akt signaling pathway-related genes in rat intestinal tissue, whereas MAGL inhibitor treatment inhibits the expression of related pathway genes in SAP rats. Focal adhesions have important functions in the intestinal epithelial barrier (Ma et al., 2013; Khan et al., 2014). The PI3K-Akt signaling pathway is a critical signaling pathway affected by various drugs in the treatment of intestinal mucosal barrier injury (Zhuang et al., 2019; Liu et al., 2020; Piao et al., 2020).

Among the DEGs verified by RT-qPCR, PAK2 is a p21 protein-activated kinase that inhibits PAK2 activity and immune responses (Ogura et al., 2016) and mediates the cellular response to oxidative stress-induced apoptosis (Huang et al., 2020). Xiap inhibits apoptosis. Knocking out or inhibiting Xiap expression changes cell death from necrosis to apoptosis, reduces the inflammatory response, and effectively reduces the severity of SAP (Liu et al., 2017). Kitlg encodes the ligand of c-Kit, a receptor tyrosine kinase, and participates in many biological processes that include hematopoiesis, gametogenesis, and melanogenesis (Talenti et al., 2018).

Abnormal ASEs of RNA play a major role in human diseases (Scotti and Swanson, 2016). AS plays a major role in the occurrence and development of intestinal tumors (Yuan et al., 2017; Wu et al., 2020). At present, the function of AS in the intestinal barrier has not been reported. On the basis of RNA-sequencing data, overlap analysis showed that 97 ASEs were downregulated in the JZL184 group compared with the SAP group, but were upregulated in the SAP group compared with the control group. Among them, Myo9b is a motor protein with a Rho GTPase activation domain (RhoGAP), which encodes myosin Ixb (Chandhoke and Mooseker, 2012). Myo9b-deficient mice show impairment of the intestinal barrier function and superficial ileal ulcers (Hegan et al., 2016). Lsp1 is a leukocyte-specific protein that forms a bimolecular protein complex with myosin 1e and regulates adhesion kinetics and cell migration (Schäringer et al., 2021). Git2 is a G protein-coupled receptor kinase-interacting factor involved in regulating thymocyte positive selection, neutrophil direction perception, and cell motility in the immune response and inhibits colitis by negatively regulating Toll-like receptor signaling (Wei et al., 2014).

RNA-binding proteins (RBPs) are central for post-transcriptional regulation of gene expression by interacting with specific sequences or structural elements in their target transcripts (Licatalosi and Darnell, 2010). RBPs have important functions in ASE regulation (Fu and Ares, 2014). It is necessary to further analyze whether there is abnormal expression of RBPs in the intestines of SAP rats resulting in changes in the AS pattern of a large number of genes. A total of 132 RBPs were screened by crossing the abnormally expressed genes we identified with the reported RBP genes, and the top 10 RBPs were extracted, which included HNRNPDL, ZCRB1, CIRBP, NUPL2B, SRFBP1, MT1, GNL2, TATDN2 and USP3. Among them, HNRNPDL coexpressed the most variable shear events as the main RBP. Hnrnpdl protein is an RNA-binding protein involved in post-transcriptional AS regulation (Li et al., 2019). The presence of the exon that corresponds to its PrLD is responsible for the incorporation of HNRNPDL into multivalent hnRNP assemblies that, in turn, control the AS of other genes (Gueroussov et al., 2017). We verified that the ratio value of ASEs-Kcnn4 and sp140 had the opposite trend with the expression of Hnrnpdl.

There are some limitations of our approach in this study. The aim of this study is to globally explore the transcriptome changes of JZL184 effects. Even these large datasets may provide a plenty of information, the causal relationship between changes in these genes/pathways and the SAP pathogenesis/JZL184 effects is still not clear. So, further studies are now carrying out to investigate the underlying mechanisms by focusing on selected proteins and pathways.

In conclusion, MAGL inhibition improved intestinal mucosal barrier injury in SAP rats and induced a large degree of differential gene expression and alternative splicing events. RNA-binding protein HNRNPDL might play an important role in improving intestinal mucosal barrier injury by affecting alternative splicing. Therefore, targeting MAGL is a promising therapeutic approach for the treatment of SAP.

## Data Availability

Data available within the article or its supplementary materials. The sequencing data presented in the study are deposited in the GEO database repository, accession number GSE197930.

## Abbreviations

2-AG, 2-arachidonoylglycerol; AS, alternative splicing; ASEs, alternative splicing events; AEA, anandamide; CB1, cannabinoid receptor 1; DEG, differentially expressed gene; FD4, fluorescein isothiocyanate-dextran; GO, gene gntology; HE, hematoxylin and eosin; HPLC-MS, high-performance liquid chromatography-mass spectrometry; IL-6, interleukin-6; IECs, intestinal epithelial cells; KEGG, kyoto encyclopedia of genes and genomes; MAGL, monoacylglycerol lipase; PBS, phosphate-buffered saline; PCA, principal component analysis; RASE, regulated alternative splicing event; RBP, RNA-binding protein; RT-qPCR, real-time Quantitative Reverse Transcription; SAP, severe acute pancreatitis; TPM, tags per million; TEM, transmission electron microscopy.
