# Corrigendum: Effect of Monoacylglycerol Lipase Inhibition on Intestinal Permeability of Rats With Severe Acute Pancreatitis

**DOI:** 10.3389/fphar.2022.965348

**Published:** 2022-08-08

**Authors:** Jing Wang, Hongwei Xu, Tianjie Chen, Changqin Xu, Xiaohua Zhang, Shulei Zhao

**Affiliations:** Shandong Provincial Hospital Affiliated to Shandong First Medical University, Jinan, China

**Keywords:** monoacylglycerol lipase, severe acute pancreatitis, intestinal permeability, differentially expressed genes, alternative splicing events, RNA-binding protein

In the published article, an author name was incorrectly written as Shule Zhao. The correct spelling is Shulei Zhao.

The authors apologize for this error and state that this does not change the scientific conclusions of the article in any way. The original article has been updated.

## Publisher’s Note

All claims expressed in this article are solely those of the authors and do not necessarily represent those of their affiliated organizations, or those of the publisher, the editors and the reviewers. Any product that may be evaluated in this article, or claim that may be made by its manufacturer, is not guaranteed or endorsed by the publisher.

