# Supplementary material for: Effect of Monoacylglycerol Lipase Inhibition on Intestinal Permeability of Rats With Severe Acute Pancreatitis
Source: Front Pharmacol. 2022 Apr 12;13:869482. doi: 10.3389/fphar.2022.869482 (PMC9039313; doi:10.3389/fphar.2022.869482)
Supplement: Supplementary file 1 [file Table1.docx]

**Table S1 Primers for qRT-PCR**

| Chuk-F | AGAACTCGGTCCTGACTC |
| --- | --- |
| Chuk-R | GGGAAAGAGGCAGAAAGAA |
| Hnrnpdl -F | AAGCAGATACCATCAGATAGG |
| Hnrnpdl -R | CTAGCACCACCTCTTCCA |
| Itga2 -F | CTGCTGAGGACGATGATG |
| Itga2 -R | CACTAAACGGAAGACAGGAT |
| Kitlg -F | TTAGGCAGTTAGGTGTAGGT |
| Kitlg -R | AACAGCAAGGACTTCTCATT |
| Pak2 -F | CGCAGTACTATGGTTGGAA |
| Pak2 -R | TGAGGTATGGAGGCTCTC |
| Rock1 -F | TGACTGACAGGCATTTCC |
| Rock1 -R | AAGCACACTGAGGATTGTT |
| Rock2 -F | TTGGATGCTGTTCGTGTAT |
| Rock2 -R | TTCTCTATGGTCATGTGGTT |
| Tnc -F | GGACTGGTTGTATTGATGCT |
| Tnc -R | TTGCGGAGATGAAACTAAGA |
| Xiap -F | AGGAAGTAGATAGGAGAATGTC |
| Xiap -R | TGTGAGTGTAAGTGGATACC |
| Lsp1-M-F | AGCTGTTGAGGCTCACAACT |
| Lsp1-AS-F: | CAAGAACCAGGCTCACAACT |
| Lsp1-M/AS-R: | CTGGCTGTTCCAGGAAATGG |
| Kcnn4-M/AS-F: | ACTGGCATCGGACTCATGGTG |
| Kcnn4-AS-R | TACAGCACCCACTTGCAACCC |
| Kcnn4-M -R: | CATTGACAGCCTTGCAACCC |
| Eif5a -M/AS-F | GCTGAGCACTGCATTGGGAAGG |
| Eif5a -AS-R | TCATCCCGCAGTTGGAATCGA |
| Eif5a -M -R | CCAGCCAGATTTGGAATCGA |
| Commd1-M/AS-F | TGGCAAAGGCACCCACGGAACA |
| Commd1-AS-R | GAAGTGGACGTCCATCTTTC |
| Commd1-M -R | ATTCAAATACTCCATCTTT |
| Git2-M/AS-F | TGACCAGCCAGACTATGACA |
| Git2-AS-R | AGAGTTTGAAGCTTCTGCCGGT |
| Git2-M -R | GAATCTAGGCCTTCTGCCGGT |
| Myo9b -M/AS-F | TCTGACGAGGAGAACCTTGACT |
| Myo9b -AS-R | GTGTCTTCACCTTCTGCGGC |
| Myo9b -M -R | TGCGGGGGGCCCTTCTGCGGC |
| Sp140-M/AS-F | GAGCACAACAACCCACAAGT |
| Sp140 -AS-R | TTCTTTTTTCCTCCTTGACT |
| Sp140 -M -R | AGTGTCCATGCTCCTTGACT |
