# Supplementary figures and images for: Effect of Monoacylglycerol Lipase Inhibition on Intestinal Permeability of Rats With Severe Acute Pancreatitis
Source: Front Pharmacol. 2022 Apr 12;13:869482. doi: 10.3389/fphar.2022.869482 (PMC9039313; doi:10.3389/fphar.2022.869482)

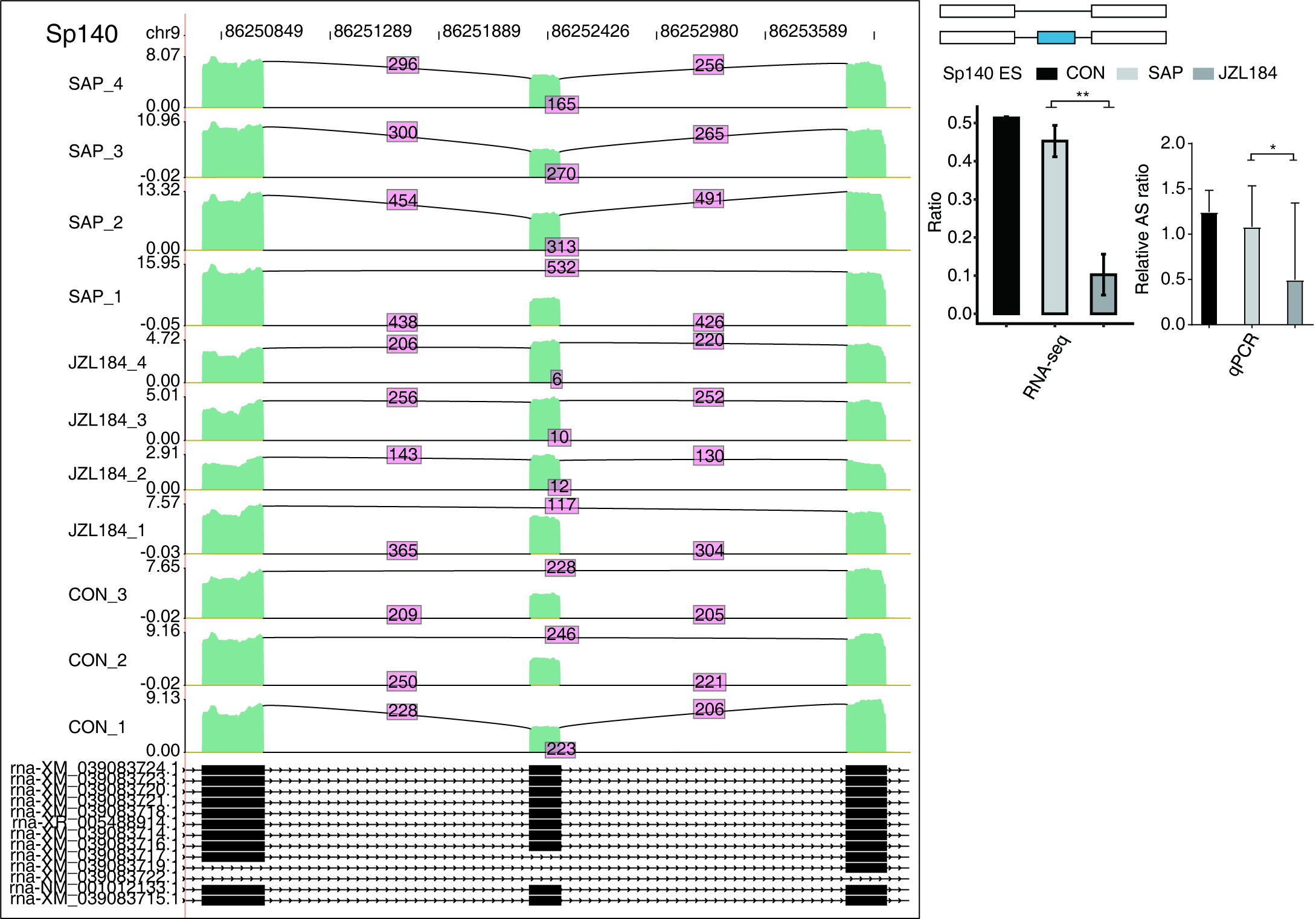

Supplement: Supplementary file 2 [file Image2.tif]

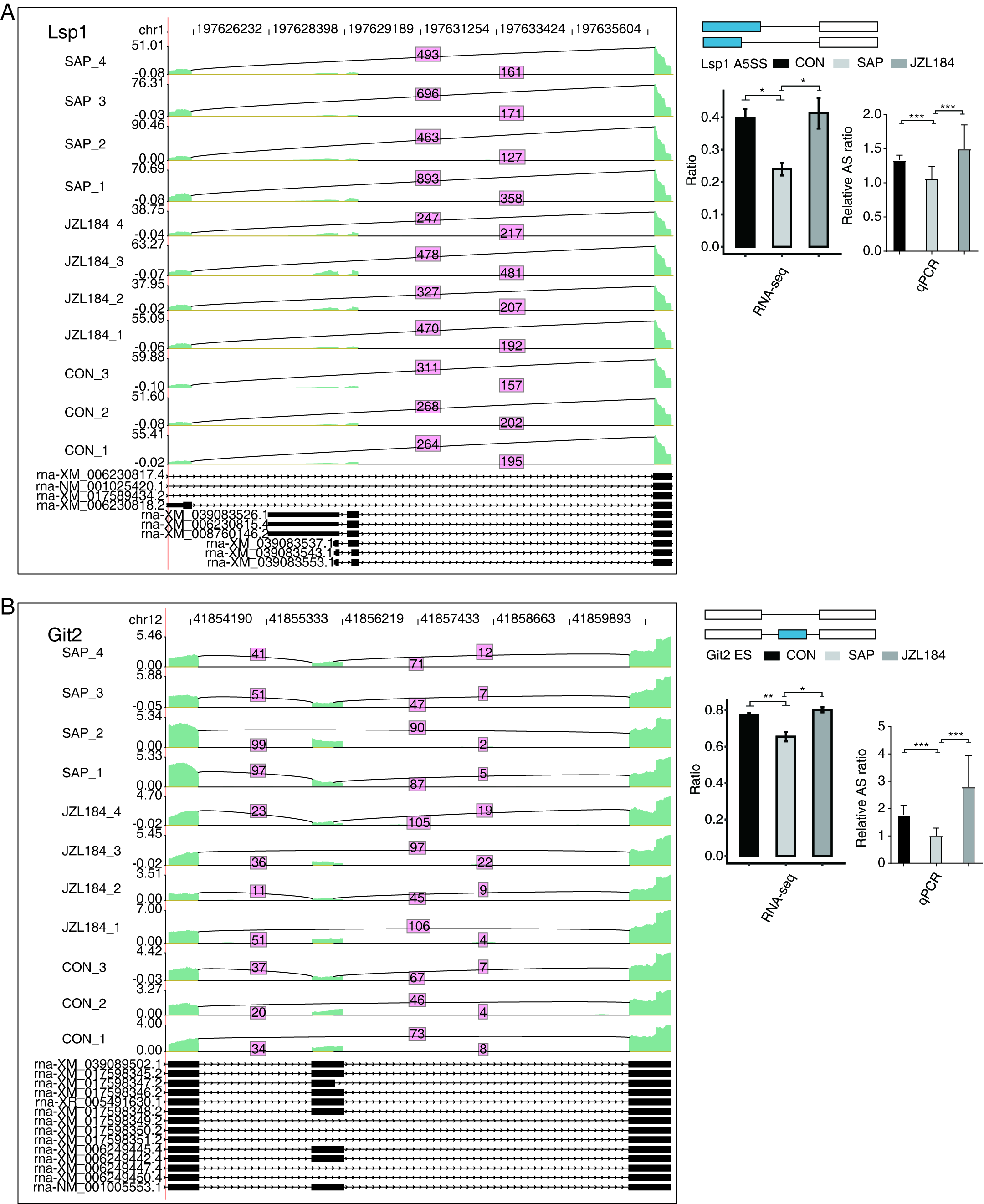

Supplement: Supplementary file 3 [file Image1.tif]
