# Supplementary material for: Effect of Monoacylglycerol Lipase Inhibition on Intestinal Permeability of Rats With Severe Acute Pancreatitis
Source: Front Pharmacol. 2022 Apr 12;13:869482. doi: 10.3389/fphar.2022.869482 (PMC9039313; doi:10.3389/fphar.2022.869482)
Supplement: Supplementary file 4 [file DataSheet1.docx]

**Supplementary Figure Legends:**

**Figure.S1 MAGL regulates alternative splicing of genes, including Lsp1 (A) and Git2(B).**

1. MAGL regulates alternative splicing of Lsp1. **Left panel**: IGV-sashimi plot showing the regulated alternative splicing events and binding sites across mRNA. Reads distribution of RASE is plotted in the up panel and the transcripts of each gene are shown below. **Right panel**: The schematic diagrams depict the structures of ASEs. RNA-seq validation of ASEs are shown at the bottom of the right panel. Error bars represent mean ± SEM. *** P-value < 0.001, ** P-value < 0.01, * P-value < 0.05.
2. MAGL regulates alternative splicing of Git2. **Left panel**: IGV-sashimi plot showing the regulated alternative splicing events and binding sites across mRNA. Reads distribution of RASE is plotted in the up panel and the transcripts of each gene are shown below. **Right panel**: The schematic diagrams depict the structures of ASEs. RNA-seq validation of ASEs are shown at the bottom of the right panel. Error bars represent mean ± SEM. *** P-value < 0.001, ** P-value < 0.01, * P-value < 0.05.

**Figure.S2 MAGL regulates alternative splicing of genes, including Sp140 (A).**

MAGL regulates alternative splicing of Sp140. **Left panel**: IGV-sashimi plot showing the regulated alternative splicing events and binding sites across mRNA. Reads distribution of RASE is plotted in the up panel and the transcripts of each gene are shown below. **Right panel**: The schematic diagrams depict the structures of ASEs. RNA-seq validation of ASEs are shown at the bottom of the right panel. Error bars represent mean ± SEM. *** P-value < 0.001, ** P-value < 0.01, * P-value < 0.05.
